# One-lung ventilation using a laryngeal mask airway and bronchial blocker in a patient with vocal cord cancer: a case report

**DOI:** 10.1186/s40981-022-00513-8

**Published:** 2022-03-17

**Authors:** Kenichi Takechi, Yoko Sanki, Kei Abe, Ichiro Shimizu

**Affiliations:** Department of Anesthesia, Matsuyama Red Cross Hospital, 1 Bunkyocho, Matsuyama City, Ehime Japan

**Keywords:** One-lung ventilation, Vocal cord cancer, Laryngeal mask airway, Bronchial blocker

## Abstract

**Background:**

One-lung ventilation is a standard technique for thoracic anesthesia. Usually, one-lung ventilation requires a large-bore tracheal tube. Therefore, in patients with vocal cord morbidity, it is challenging to achieve one-lung ventilation while preventing the damage of vocal cord lesions.

**Case presentation:**

A 77-year-old man was diagnosed with vocal cord cancer and lung tumor. One-lung ventilation with a combination of a laryngeal mask airway and bronchial blocker was planned to avoid unexpected vocal cord injury. After securing the airway with a laryngeal mask airway, a bronchial blocker was placed under fiberscope guidance. The bronchial blocker passed through a position far enough from the vocal cord lesion. The bronchial blocker provided a clear view of the operative field. The patient’s perioperative course was uneventful.

**Conclusions:**

When one-lung ventilation is required for patients with vocal cord lesions, a combination of a laryngeal mask airway and bronchial blocker is considered a good option.

## Background

One-lung ventilation is a standard technique used to administer thoracic anesthesia. There are several methods for achieving one-lung ventilation, including the use of a specially designed tracheal tube, such as a double-lumen tube, or the attachment of a bronchial blocker to a single-lumen tracheal tube [1]. Typically, both methods require the use of a large-bore tracheal tube. Therefore, in patients with vocal cord morbidity, especially in patients with vocal cord cancer, it is challenging to achieve one-lung ventilation while preventing the damage of vocal cord lesions. Another reported method for achieving one-lung ventilation is through a combination of a laryngeal mask airway and a bronchial blocker [2, 3]. However, which cases should undergo one-lung ventilation through the combined use of a laryngeal mask airway and a bronchial blocker is still controversial. We used a combination of a laryngeal mask airway and bronchial blocker to achieve one-lung ventilation in patients with vocal cord cancer to avoid using large-bore tracheal tubes.

This manuscript adheres to the applicable EQUATOR guidelines. The patient provided written informed consent for the publication of this case report.

## Case presentation

A 77-year-old man (American Society of Anesthesiologists grade 3) who underwent microscopic laryngeal surgery for vocal cord tumor and leukoplakia was diagnosed with vocal cord squamous cell carcinoma. In addition, preoperative computed tomography revealed a lung tumor that was suspected to be malignant. After mutual discussion between the thoracic surgeon and the otolaryngologist, they decided to perform a left lower lung lobectomy before administering additional treatment for vocal cord cancer.

The patient had a history of hypertension and diabetes, which were controlled with medication. Two weeks before thoracic surgery, laryngoscopy was performed by an otolaryngologist, which revealed an elevated lesion on the vocal cord and mucosal thickening. After a preoperative conference between the anesthesiologist and thoracic surgeon, one-lung ventilation with a combination of a laryngeal mask airway and bronchial blocker was planned to avoid unexpected vocal cord injury and tumor dislodgment due to the insertion of a large-bore tracheal tube.

A standard anesthetic protocol was implemented, which involved routine noninvasive arterial blood pressure monitoring, electrocardiography, and oxygen saturation measurement on arrival to the operating room; the patient’s vital signs were stable. Before the induction of general anesthesia, a thoracic epidural catheter was inserted into the 6th–7th thoracic interspace. Anesthesia was induced using propofol (2 mg/kg), remifentanil (0.15 μg/kg/min), and rocuronium (0.8 mg/kg) and was maintained using desflurane (4%), remifentanil (0.1–0.15 μg/kg/min), and rocuronium (6 μg/kg/min). Continuous thoracic epidural anesthesia with 0.2% ropivacaine was used for maintaining intra- and postoperative analgesia. Before making a skin incision, cefazolin (1 g) was administered, followed by additional doses every 3 h. After the induction of anesthesia, a 22-G catheter was inserted into the right radial artery for blood sampling and continuous blood pressure monitoring.

**Fig. 1:**
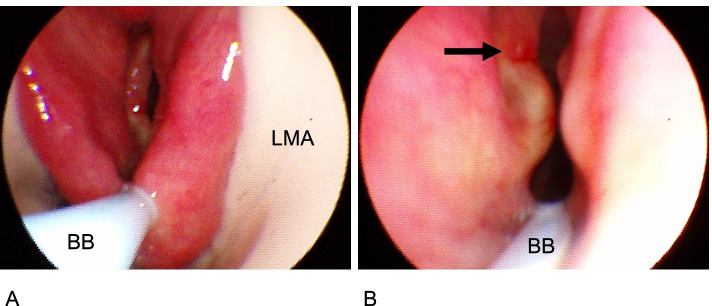
**A** General view of laryngoscopy. BB, bronchial blocker; LMA, laryngeal mask airway. **B** Laryngoscopy of vocal cord lesion. Black arrow, an elevated lesion on the vocal cord. The bronchial blocker passes through a position far enough from the vocal cord lesion

After inducing anesthesia and securing the airway with a laryngeal mask airway (ProSeal Laryngeal mask airway #4, Teleflex, Ireland), a 9-Fr bronchial blocker (Phycon TCB bronchial blocker, Fuji Systems, Japan) was connected to the laryngeal mask airway via the multiport adaptor. The laryngeal mask airway cuff was inflated with 10 ml air. The blocker was placed in the left main bronchus under the guidance of a fiberscope 3.4 mm in diameter. The bronchial blocker passed through a position far enough from the vocal cord lesion (Fig. 1). The one-lung ventilation was started immediately after the patient was placed in the right lateral decubitus position. The bronchial blocker balloon was inflated with 6 ml air. During one-lung ventilation, the following ventilator settings were used: pressure control ventilation; inspirated pressure, 20 cm H_2_O; inspired O_2_ fraction, 0.6–1.0; and inspiratory fresh gas flow, 2 l/min. The respiratory rate was adjusted to 12–18 breaths/min to maintain an end-tidal carbon dioxide pressure of 35–45 mmHg. The surgery was initiated 25 min post the one-lung ventilation, and the lung collapse caused by the bronchial blocker provided a clear view of the operative field. There was no audible peri-laryngeal leakage during one-lung ventilation.

The surgery was started thoracoscopically and was converted to open thoracotomy midway through the procedure. The operation time was 378 min, and the one-lung ventilation time was 350 min. After the one-lung ventilation was completed, tracheal suctioning was performed using a fiberscope to avoid postoperative atelectasis, but the quantity of sputum was small. After two-lung ventilation, the lung expanded normally. After the surgery was completed and the patient was awake, the laryngeal mask airway was removed. The patient was transferred to the intensive care unit for postoperative care. The patient’s postoperative course was uneventful, and he was discharged without complications.

## Discussion

One-lung ventilation is a technique used during lung resection surgery to facilitate optimal surgical conditions. The double-lumen tube is the most widely used device for achieving safe one-lung ventilation. For male and female Asian patients, double-lumen tube sizes of 39 Fr and 35 Fr, respectively, are usually chosen [4]. The transverse diameters of the 39-Fr and 35-Fr double-lumen tubes are approximately 14 mm and 13 mm, respectively. Thus, physical stimulation caused by a double-lumen tube to the vocal cord is considered to be greater than that caused by a normal single-lumen tracheal tube [5]. Another technique to achieve one-lung ventilation is the combination of a single-lumen tracheal tube and a bronchial blocker. As a bronchial blocker, the use of an ≧ 8.0-mm tracheal tube is recommended [1]. In contrast, laryngeal microsurgery for vocal cord cancer is usually performed using a small-bore tracheal tube [6]. This not only ensures ease of operation but also helps avoid the dislodgement and injury of the tumor by tracheal intubation [7]. The tip of a laryngeal mask airway is located in the posterior lamina of the cricoid and does not pass through the glottis. Therefore, in the current case, we performed one-lung ventilation with a combination of a laryngeal mask airway and a bronchial blocker to avoid inserting a large-bore tracheal tube.

One-lung ventilation with a combination of laryngeal mask airway and bronchial blocker has been initially reported in cases of difficult tracheal intubation in adults [8, 9]. Because they were able to guide the bronchial blocker into the trachea, in those cases, it would have been a more secure method to guide the tube exchanger through the laryngeal mask airway, exchange to the single-lumen large-bore tracheal tube, and use the bronchial blocker through the tracheal tube. Thus, even if reports declare that it is a safe method, which cases should undergo one-lung ventilation with the combination of laryngeal mask airway and bronchial blocker is still controversial. In our case, the vocal cord cancer was partially resected by laryngeal microsurgical biopsy, but the presence of an elevated lesion was still confirmed by preoperative and intraoperative laryngoscopy. We believe that a good indication for the use of a combination of a laryngeal mask airway and a bronchial blocker is the necessity for one-lung ventilation in patients with vocal cord lesions.

It has been reported that a combination of a laryngeal mask airway and a bronchial blocker is appropriate for short-term thoracic surgery [2]. One of the differences between using a laryngeal mask airway and a tracheal tube is the simplicity of tracheal suctioning. In particular, when the one-lung ventilation time is prolonged, as in this case, it is necessary to perform tracheal suction using a fiberscope after resuming two-lung ventilation to prevent postoperative atelectasis.

Airway securement using a laryngeal mask airway is associated with several complications, including edema of the glottis, which may obstruct the airway [10]. In case the vocal cord tumor is large and airway stenosis or ventilation cannot be achieved due to air leaks around the laryngeal mask airway, this method is not considered feasible. In such cases, the use of a small-bore tracheal tube and a bronchial blocker passing next to the tracheal tube has been reported [11]. If ventilation becomes inadequate during surgery using laryngeal mask airway, a small-bore tracheal tube can be inserted via the laryngeal mask airway using the guidance of a fiberscope.

## Conclusion

In conclusion, we used a laryngeal mask airway and a bronchial blocker for one-lung ventilation in a patient with vocal cord cancer. When one-lung ventilation is required for patients with vocal cord lesions, this method is considered a good option. Therefore, experience with this method is necessary for anesthesiologists.

## Data Availability

Not applicable.
